# The relationship between quality of life, sleep quality, mental health, and physical activity in an international sample of college students: a structural equation modeling approach

**DOI:** 10.3389/fpubh.2024.1397924

**Published:** 2024-07-10

**Authors:** Imen Moussa-Chamari, Abdulaziz Farooq, Mohamed Romdhani, Jad Adrian Washif, Ummukulthoum Bakare, Mai Helmy, Ramzi A. Al-Horani, Paul Salamh, Nicolas Robin, Olivier Hue

**Affiliations:** ^1^Physical Education Department, College of Education, Qatar University, Doha, Qatar; ^2^Laboratoire ACTES, UFR-STAPS, Université des Antilles, Pointe-à-Pitre, France; ^3^Aspetar, Orthopaedic and Sports Medicine Hospital, FIFA Medical Centre of Excellence, Doha, Qatar; ^4^Interdisciplinary Laboratory in Neurosciences, Physiology and Psychology: Physical Activity, Health and Learning (LINP2), UFR STAPS (Faculty of Sport Sciences), Paris Nanterre University, Nanterre, France; ^5^Physical Activity, Sport and Health, UR18JS01, National Observatory of Sports, Tunis, Tunisia; ^6^Sports Performance Division, Institut Sukan Negara Malaysia (National Sports Institute of Malaysia), Kuala Lumpur, Malaysia; ^7^Nigeria Olympic Committee, Medical and Scientific Commission, Lagos, Nigeria; ^8^Psychology Department, College of Education, Sultan Qaboos University, Muscat, Oman; ^9^Department of Sports Sciences, Yarmouk University, Irbid, Jordan; ^10^College of Health Sciences, University of Indianapolis, Indianapolis, IN, United States

**Keywords:** structural equation model, well-being, higher education, mental health, sleep behavior

## Abstract

**Objective:**

We assessed the direct and indirect relationships between sleep quality, mental health, and physical activity with quality of life (QOL) in college and university students.

**Methods:**

In a cross-sectional design, 3,380 college students (60% females; age = 22.7 ± 5.4) from four continents (Africa: 32%; America: 5%; Asia: 46%; and Europe: 15%; others: 2%) completed the Pittsburgh Sleep Quality Index (PSQI); Insomnia Severity Index (ISI); Epworth Sleepiness Scale (ESS); the Depression, Anxiety, and Stress Scale 21 (DASS); the International Physical Activity Questionnaire short-form (IPAQ); and the World Health Organization Quality of Life-BREF (WHOQOL-Brief).

**Results:**

We showed that sleep quality, insomnia, and depression had direct negative effects on the physical domain of QOL (β = −0.22, −0.19, −0.31, respectively, *p* < 0.001). There was a strong negative direct association between depression and the psychological domain of QOL (β = −0.60, z = −22.21, *p* < 0.001). Both stress and PSQI had direct effects on social relationships QOL (β = 0.11; z = 4.09; and β = −0.13; z = −7.40, respectively, *p* < 0.001). However, depression had the strongest direct impact on social relationships QOL (β = −0.41, z = −15.79, *p* < 0.001).

**Conclusion:**

The overall QOL of university students is associated with their sleep quality, mental health, and physical activity warranting further interventional studies aiming at improving students’ quality of life.

## Introduction

The World Health Organization (WHO) defines quality of life (QOL) as “individuals’ perception of their position in life in the context of the culture and value systems in which they live and in relation to their goals, expectations, standards and concerns” (1). In recent years, the importance of QOL has been acknowledged and prioritized by the WHO and many governments across the world. This increased priority is attributed to the understanding of QOL’s link with critical health issues including chronic diseases and mental disorders (2). In particular, the QOL of university students has received an increased attention, as studies have reported that a substantial number of students are subjected to psychological and emotional distress and burnout. Indeed, University students may find academia stressful because it implies several simultaneous stressors (e.g., the change in lifestyle, time management, increased responsibility, financial stress) with no guarantee of a favorable return. Therefore, the prevalence of mental disorders, especially stress and depression, has increased dramatically in recent decades, affecting a wide range of student cohorts (3–5). A lower perception of QOL domains/subcomponents (i.e., physical, psychological, social, and environmental) may result from this pressure (5). This is concerning given the important role of well-being in academic performance and in reducing students’ dropout rates (6). Few studies have examined the connection between mental health disorders and QOL in college students, with most of the previous research focusing on medical science students (4). Moutinho et al. (3) found a high prevalence of mental health disorders among Brazilian medical students, including substantial prevalence of depression, anxiety, and stress levels, affecting their QOL. Furthermore, Freitas et al. (7) reported an association between QOL and symptoms of depression, anxiety, and stress in college students, showing that approximately 50% of the sample exhibiting mild to severe symptoms. They also reported that depressive symptoms were linked to lower overall QOL, especially in the psychological domain (7).

Facing the above mentioned myriad of stressors, university students might find their sleep compromised. Studies have shown that students could be subjected to several sleep disorders (e.g., insomnia, daytime sleepiness, non-restorative sleep) that can potentially affect their mental health, and academic performance (8, 9). University students usually sacrifice their sleep to compensate the lack of time imposed by their lifestyle (e.g., academic pressure, financial stress, screen time, social engagement). For instance, acute sleep restriction or deprivation can lead to a range of undesirable effects, from simple mood disruption (10) to more complex increase in systemic inflammation (11). When such sleep deprivation becomes chronic, it can contribute to mental health issues (12), metabolic disorders that lead to overweight and obesity (13), and/or an increased pain sensitivity (11), thereby impacting physical health perception. Furthermore, sleep deprivation can result in emotional and social issues, cognitive function impairment, reduced work and academic performance, and/or increased daytime sleepiness, among other consequences (14). Altogether, sleep deprivation/restriction affects several aspects of health, potentially affecting the QOL. However, this relationship has not been studied in university students.

Of particular interest, physical activity (PA) may play a cardioprotective role (15) and may counteract the sleep deprivation-induced cognitive degradation (16). Therefore, by engaging in regular PA, university students can experience a variety of benefits that contribute to a more positive QOL. Indeed, PA has been associated with improvements in at least one of the four QOL domains (i.e., physical, psychological, environmental health, and/or social interaction) in university students (17). Moreover, PA could reduce perceived stress, enhance well-being, and alleviate mental health symptoms (4), highlighting the importance of incorporating regular PA into the daily routines of university students to optimize their overall QOL.

The QOL of college students has been explored in the literature, most of which focusing on its’ separate domains. Our study considered a more holistic perspective to assess the relationship between QOL domains and three key factors: (i) sleep quality, (ii) psychological factors, and (iii) PA levels, in an international sample of university students. Therefore, we aimed to establish a model of relationships between QOL, sleep, mental health, and physical activity in college students using structural equation modeling.

## Materials and methods

We conducted a web-based, Google Forms® cross-sectional survey administered in three languages: Arabic, English, and French. The survey was released from 1st October 2021 to 31st of March 2022. The closing date has been chosen because it corresponded to the eve of Ramadan month for Muslims worldwide. We therefore avoided potential interferences of Ramadan observance with the study outcomes (18). We promoted the survey via (i) social-media platforms (e.g., Facebook, Instagram, and Twitter), (ii) messaging applications (e.g., Viber, WhatsApp,) and (iii) official universities websites.

A total of 3,509 survey responses were received (see Table 1). Following the process of data cleaning based on inclusion criteria: University/College students, aged 18 years or more at the time of completing the survey, not taking medication for chronic illness conditions and exclusion criteria: Individuals who self-reported having been diagnosed with chronic sleep disorders were excluded from the study, 3,380 responses of students from 49 countries were included in our analysis.

**Table 1:** Distribution of participants according to sex, age and continents.

	Frequency	Percent
**Sex**		
Female	2,037	60.27
Male	1,343	39.73
**Age**		
≤20	1,472	43.55
21–25	1,353	40.03
26–30	273	8.08
31–35	134	3.96
≥36	148	4.38
**Continent**		
Africa	1,086	32.13
America	181	5.36
Asia	1,544	45.68
Europe	523	15.47
N/A	46	1.36
Total	3,380	100

The database has previously been used to set descriptive statistics about our sample in another article currently under review. The latter study does not use any results from the present manuscript.

### Ethics

The study’s protocol was reviewed and received approval from the Ethics Committee of Qatar University (QU-IRB 1510-EA/21). The study strictly adheres to the ethical standards of the Declaration of Helsinki (2013 and its subsequent amendments). Additionally, we ensured participant’s anonymity according to the guidelines in the General Data Protection Regulation.^1^ Participation in the study was voluntary, with participants having the option to withdraw from the study at any time without facing any penalties or consequences. Before commencing the survey, participants provided online informed consent for their involvement in the survey.

### Data collection

Participation was limited to students who were aged 18 years or older at the time of completing the survey. A total of 129 individuals with self-reported diagnosis of chronic sleep disorders were excluded from the study.

### Instruments

The validated tools below have been used in Arabic, English, and French (see Supplementary Material Table S1).

### The World Health Organization Quality of Life—BREF

The World Health Organization Quality of Life (WHOQOL)-BREF comprises 26 items assessing the QOL in four domains: physical health (7 items); psychological health (6 items); social relationships (3 items); and environmental health (8 items). Each item was rated on a five-point Likert-scale, ranging from “1” (not at all/very dissatisfied) to “5” (completely/extremely/very satisfied). These ratings were then linearly converted to a scale of 0 to 100 for analysis and interpretation (2, 19, 20).

### The Pittsburgh Sleep Quality Index

The Pittsburgh Sleep Quality Index (PSQI) assesses seven key areas related to sleep patterns, including self-reported bedtimes and wake-up times, total sleep duration, time in bed, sleep efficiency, and sleep-onset latency and daytime dysfunction over the month preceding the assessment. The global PSQI score ranges from 0 to 21, where lower scores indicate better sleep quality, while higher scores of ≥5 or ≥8 indicate poor sleep quality, or very poor sleep quality, respectively (21–23).

### The Epworth Sleepiness Scale

The Epworth Sleepiness Scale (ESS) assesses excessive sleepiness due to sleep debt or clinical sleep disorders. This eight-item scale assesses how sleepy one has felt; participants indicate the likelihood that they would fall asleep while doing everyday-like activities (e.g., watching TV, sitting and talking to someone, or stopping at a traffic light), with responses from “0” (would never doze) to “3” (high chance of dozing). The ESS scores were interpreted as follows: scores (i) 0 to 5 indicate lower normal daytime sleepiness; (ii) 6 to 10: higher normal daytime sleepiness; (iii) 11 to 12: mild excessive daytime sleepiness; (iv) 13 to 15: moderate excessive daytime sleepiness, and (v) 16 to 24: severe excessive daytime (24–26).

### The Insomnia Severity Index

The Insomnia Severity Index (ISI) evaluates insomnia in adults, capturing information of the preceding month. Each item (e.g., falling asleep, staying asleep, early awakening,) in the test is measured using a 5-point Likert scale. Scores (i) ranging from 0 to 7 indicate the absence of clinically significant insomnia; (ii) 8 to 14: subthreshold insomnia; (iii) 15 to 21: moderately serious clinical insomnia, and (iv) 22 to 28: severe clinical (27–29).

### The Depression, Anxiety, and Stress Scale 21

The Depression, Anxiety, and Stress Scale 21 (DASS) is a quantitative assessment tool designed to measure distress across three dimensions: depression, anxiety, and stress. Most versions of the DASS-21 follow comparable scoring criteria, prompting individuals to assess the frequency of symptoms experienced in the preceding week using a 4-point scale, ranging from “0” (not applicable to me at all) to “3” (highly applicable to me or most of the time). The total score correspond to the sum of the three sub-scores: stress, anxiety, and depression (30–32).

### Short-version of the International Physical Activity Questionnaire

The International Physical Activity Questionnaire (IPAQ) was employed to evaluate the physical activity levels of participants. This is the short form IPAQ-SF, which consists of 7 items. It includes questions on physical activities associated with work, transportation, housework, and leisure activities “during the past seven days.” The Metabolic Equivalent of the Task (METs)-min was calculated by multiplying the duration of activity in minutes by the coefficient of the activity level (e.g., 1.3 for sitting, 3.3 for walking, 4 for moderate, and 8 for vigorous activities) (33, 34).

### Statistical analysis

The collected data were analyzed using Structural Equation Modeling (SEM), to assess the relationship between QOL and other variables based upon maximum likelihood estimation method within STATA version SE 18 (35). The SEM is a multivariate statistical analysis technique used to analyze structural relationships. This technique is the combination of factor analysis and multiple regression analysis and is used to analyze the structural relationship between measured variables and latent constructs, enabling the estimation of multiple and interrelated dependencies in a single analysis. In this type of analysis, two categories of variables are utilized: endogenous (dependent: QOL with four domains) and exogenous (independent: PSQI, ISI, anxiety, physical activity, and stress) variables.

The main outcomes of the SEM analysis are β (the sum of direct and indirect effect) and Z which is the standard score (measures exactly how many standard deviations above or below the mean a data point).

## Results

### Model 1: physical health domain of QOL

**Figure 1:**
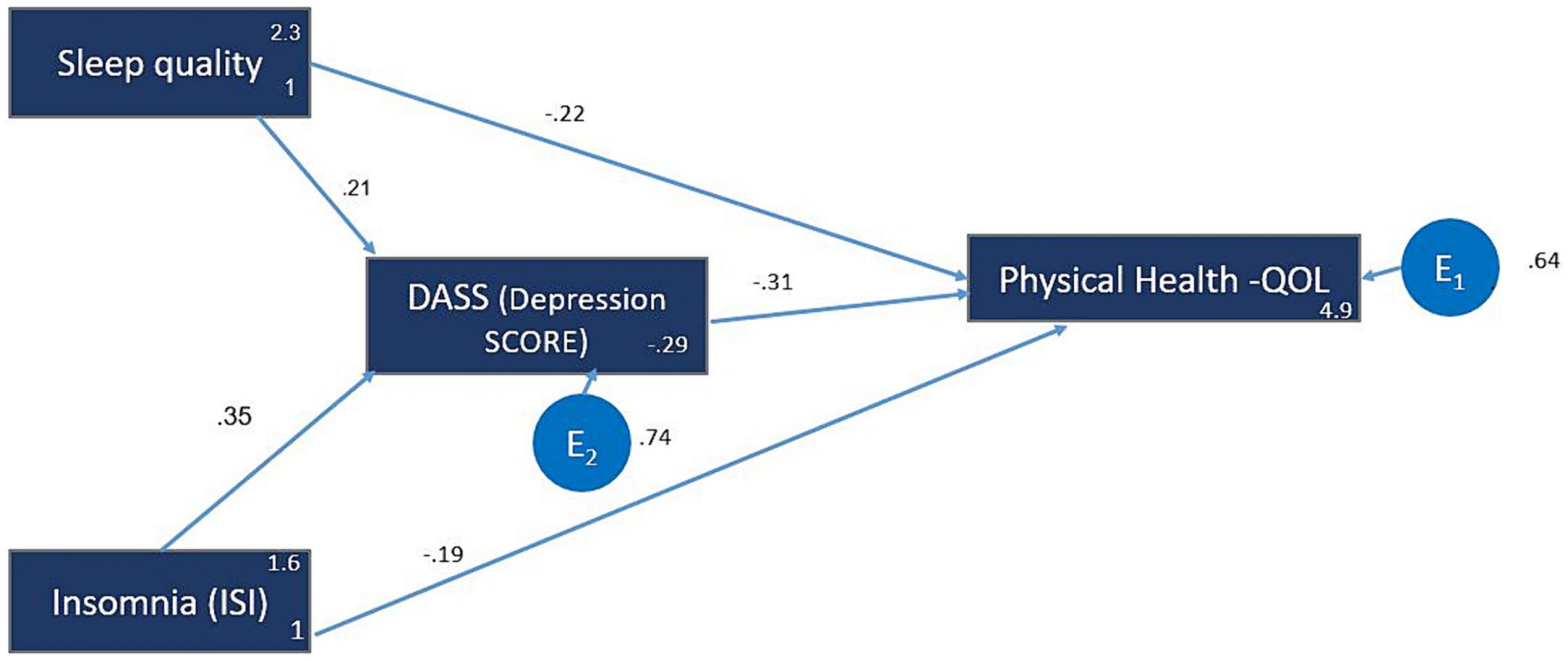
Model 1: The QOL 1 (Physical Health). The hypothesized multiple mediator models examining the relationship between sleep quality, insomnia, depression and the quality of life (QOL)—Physical health domain. The arrows represent significant relationships between two variables. The value next to the arrow represents the direct effect β standardized path coefficient (for indirect effect value *see* Table 3). The numbers next to the variables, e.g., (2, 3) and (1) for Sleep Quality, represent the average and the variance, respectively. E1 represents the error and the number next to it is the standardize relationship.

The SEM analysis revealed significant relationships of sleep quality (PSQI), ISI, and depression on the physical health domain of QOL. Sleep quality (β = 0.21; z = 10.95; *p* < 0.001) and insomnia severity (β = 0.35; z = 19.19, *p* < 0.001) were two main predictors of depression score. DASS score was the strongest standardized predictor of the physical health QOL (β = −0.31; z = −20.11; *p* < 0.001) followed by sleep quality (β = −0.22; z = −12.05; *p* < 0.001) and insomnia (β = −0.19; z = −10.08; *p* < 0.001). The reported R^2^ for the outcome variable was 35.5%. Further upon model diagnosis we discovered that root mean square error was <0.05 and comparative fit index (CFI) was greater than 0.90. Next, we examined the direct and indirect effects of each predictor. In addition to the direct effects of PSQI on QOL, there was an indirect standardized effect (β = −0.07; z = −9.42; *p* < 0.001) accounting for 23.1% of the total effect. However, insomnia had a higher indirect effect on QOL physical health (β = −11; z = −13.31; *p* < 0.001) that accounted for 36.9% of the total observed effect (Table 2). Figure 1 presents the SEM analysis of independent variables (PSQI, ISI, and depression) on the dependent variable (QOL physical health).

**Table 2:** Standardized total, direct, and indirect effects of sleep quality, insomnia, and depression on quality of life (physical health domain).

QOL1	Total β	*p*-value	Indirect β	*p*-value	Direct β	*p*-value
PSQI	−0.28	<0.001	−0.07 (23.1%)	<0.001	−0.22 (76.9%)	<0.001
Insomnia	−0.30	<0.001	−0.11 (36.9%)	<0.001	−0.19 (63.1%)	<0.001
Depression	−0.31	<0.001			−0.31 (100.0%)	<0.001

QOL 1, Quality of life (Physical health domain); PSQI, Pittsburgh Sleep Quality Index. The percentage values represent percentage of the direct effects compared to indirect effects.

### Model 2: psychological health domain of QOL

**Figure 2:**
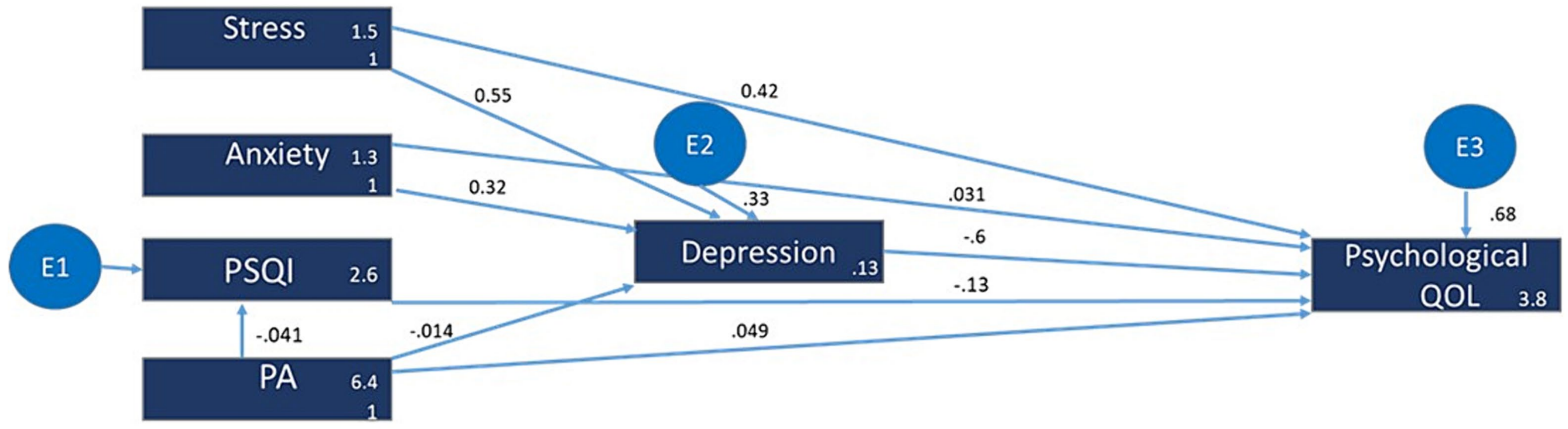
Model 2: The QOL 2 (psychological health). The hypothesized multiple mediator models examining the relationship of sleep quality, stress, depression, anxiety and physical activity and the QOL—Psychological health domain. For more figure legend details see Figure 1.

The predictors for depression were stress (β = 0.55; z = 34.83; *p* < 0.001) and anxiety (β = 0.32; z = 19.04; *p* < 0.001). However, the variable physical activity did not show any significant relationship (β = −0.01, z = −1.36, *p* = 0.17). For Depression, the R^2^ value was 0.675, indicating that approximately 67.5% of the variance in the observed depression scores can be explained by the fitted model. Depression exhibited a substantial negative impact (β = −0.60; z = −23.94; *p* < 0.001) on the psychological QOL. The PSQI showed a negative impact on the psychological QOL (β = −0.13; z = −7.09; *p* < 0.001). PA demonstrated a positive relationship (β = 0.05; z = 3.19; *p* = 0.001) on the psychological QOL. R^2^ value (0.316) indicates a moderate level of explanatory ability (Figure 2).

There was a strong indirect influence of stress on the psychological QOL (β = −0.33; z = −18.31; *p* < 0.001). However, when considering a mediating variable, the overall relationship between stress and the psychological QOL becomes negative. This suggests that the mediating variable associated with stress has a counteracting effect, potentially mitigating the positive impact observed in the direct relationship.

There was an indirect influence of anxiety on the psychological QOL (β = −0.19; z = −14.26; *p* < 0.001). Similarly, to stress, when considering a mediating variable, the overall relationship between anxiety and the psychological QOL became negative. This indicates that the mediating variable associated with anxiety may attenuate the positive direct effect.

The positive indirect effect (β = 0.01; z = 2.04; *p* = 0.042) suggests a minor indirect influence of physical activity on the psychological QOL which represented only 16.7% of the total observed effect (Table 3).

**Table 3:** Standardized total, direct, and indirect effects of psychological health (quality of life) on core variables.

QOL 2	Total β	*p*-value	Indirect β	*p*-value	Direct β	*p*-value
PSQI	−0.13	<0.001	0.00	<0.001	−0.13	<0.001
Depression	−0.60	<0.001	0.00	<0.001	−0.60	<0.001
Stress	−0.29	<0.001	−0.33	<0.001	0.04	<0.001
Anxiety	−0.16	<0.001	−0.19	<0.001	0.03	<0.001
Physical Activity	0.06	<0.001	0.01	<0.001	0.05	<0.05

QOL 2, Psychological health; PSQI, Pittsburgh Sleep Quality Index.

### Model 3: social relationship domain of QOL

Both depression (β = −0.408; z = −15.79; *p* < 0.001) and PSQI (β = −0.133; z = −7.40; *p* < 0.001) exhibited negative impacts, indicating that increases in either depression or PSQI scores correspond to considerable reductions in social relationships QOL. Conversely, stress demonstrated a positive effect (β = 0.113; z = 4.09; *p* < 0.001), signifying that heightened stress levels were related with increased social relationships QOL.

Moreover, the variance explained (R^2^ values), shows that depression accounted for 64.5% of the variance in social relationships QOL, while stress and PSQI contributed to 22.3% and 15.6%, respectively. This suggests that among the studied variables, depression has the strongest explanatory power in predicting social relationships QOL.

**Figure 3:**
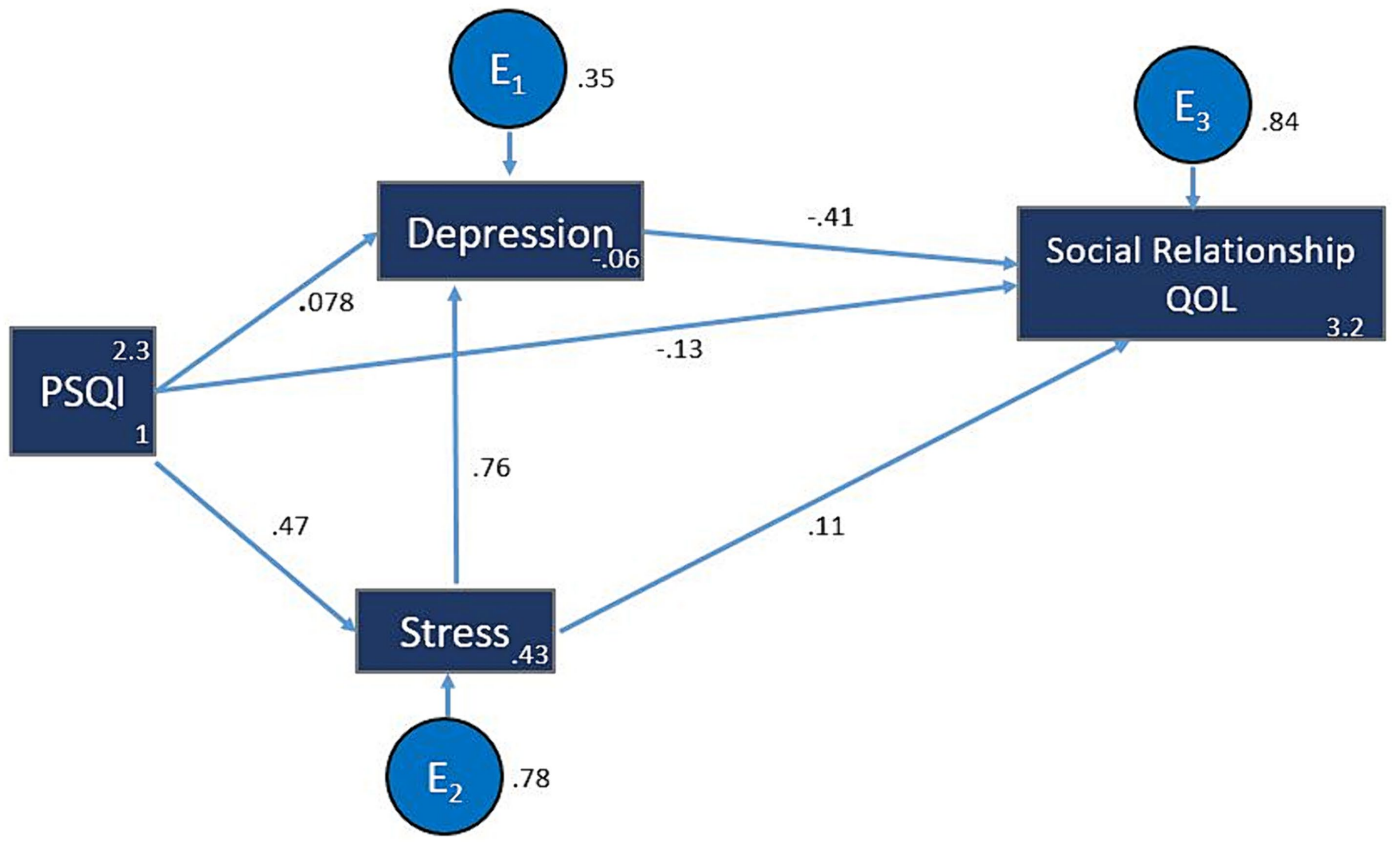
Model 3: QOL3 (Social relationship). The hypothesized multiple mediator models examining the relationship of sleep quality, stress and depression to QOL (Social relationships). For more figure legend details see Figure 1. PSQI, Pittsburgh Sleep Quality Index; QOL, Quality of Life.

The association between stress and depression was strong (β = 0.764; z = 88.10; *p* < 0.001), underlining the substantial influence exerted by stress on depression. Additionally, PSQI had a direct and robust impact on stress (β = 0.473; z = 37.40; *p* < 0.001), indicating its considerable influence on stress levels (Figure 3; Table 4).

**Table 4:** Standardized total, direct, and indirect effects of social relationships quality of life on core variables.

QOL3 Effects	Total β	*p*-value	Indirect β	*p*-value	Direct β	*p*-value
Depression	−0.41	< 0.001	(no path)	-	−0.41	<0.001
Stress	−0.2	< 0.001	−0.31	< 0.001	0.11	<0.001
PSQI	−0.26	< 0.001	−0.13	< 0.001	−0.13	<0.001

QOL3, Social Relationships; PSQI, Pittsburgh Sleep Quality Index.

### Model 4: environmental domain of QOL

**Figure 4:**
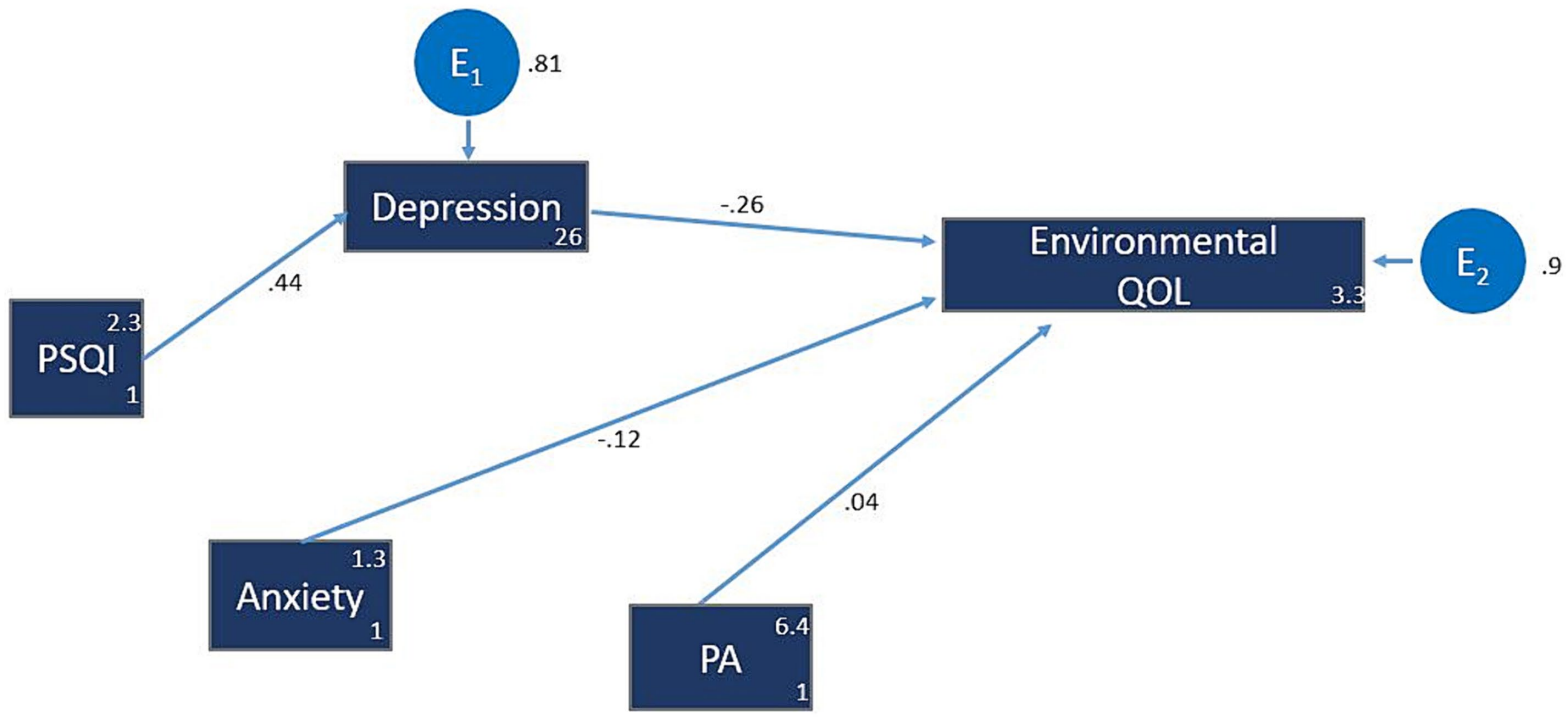
Model 4: QOL4 (Environmental domain). The hypothesized multiple mediator models examining the relationship of sleep quality, depression, anxiety and physical activity to QOL (Environmental domain). For more figure legend details see Figure 1. PSQI, Pittsburgh Sleep Quality Index; PA, Physical activity.

Depression demonstrated a substantial negative impact (β = −0.26; z = −10.21; *p* < 0.001) on the environmental QOL, indicating that for every one-unit increase in depression, QOL (environmental domain) decreased by 0.26 standard deviation. Similarly, anxiety exhibited a negative effect (β = −0.12; z = −4.60; *p* < 0.001) on the environmental QOL. Conversely, both anxiety (β = −0.12; z = −9.31; *p* < 0.001) and PA (β = 0.04; z = 2.25; *p* = 0.024) demonstrated small, yet significant impacts on environmental QOL (Figure 4; Table 5).

**Table 5:** Standardized total, direct, and indirect effects of environmental quality of life on core variables.

QOL 4 Effects	Total β	*p*-value	Indirect β	*p*-value	Direct β	*p*-value
Depression	−0.26	0.001	No path	-	−0.26	0.001
Anxiety	−0.12	0.001	No path	-	−0.12	0.001
PSQI	−0.44	0.001	−0.12	0.001	No path	
PA	0.04	0.024	No path	-	0.04	0.024

QOL 4, Quality of life (Environmental domain); PSQI, Pittsburgh Sleep Quality Index; PA, Physical activity.

## Discussion

Our findings showed that in college students: (i) QOL physical health domain was positively impacted by good sleep, and negatively affected by insomnia, and depression; (ii) psychological QOL was negatively affected by stress, anxiety and poor sleep; (iii) social relationships QOL was strongly and negatively associated with depression, and poor sleep; and (iv) environmental QOL was positively impacted by physical activity and negatively affected by depression, and anxiety.

### Physical health QOL

There are direct and indirect effects of sleep quality, insomnia and depression on the physical health QOL in college students. This aligns with previous studies that were conducted among the general population. Overall sleep quality was indirectly connected to physical and mental health domains of QOL through symptoms of insomnia in college students (36, 37). Moreover, lower sleep quality was associated with negative mood, lower self-esteem and satisfaction with physical well-being and higher daytime sleepiness (36, 37). It is noteworthy to mention that sleep quality had the stronger impact on mood and physical well-being than sleep duration (37). Although not investigated in our study, a better physical health QOL was associated to better diet and healthier body weight (38). Contrarily to earlier reports, we did not observe any association between the level of PA and physical health QOL (38).

Regular PA, even at low-to-moderate intensities, seem to be helpful in reducing depression symptoms and perceived stress (4). The detrimental effects of poor physical health and sleep quality may be reflected in the psychological and social adaptation challenges experienced by university students (39).

### Psychological health QOL

We showed that stress and anxiety scores directly and indirectly influenced psychological health domain of QOL (through higher depression scores). This finding aligns with earlier reports showing an association between lower mental health and lower psychological QOL (7, 40, 41). Several studies used mindfulness therapy to reduce negative feelings (i.e., anxiety, despair, and perceived stress) in college students reporting positive results (39, 42, 43). Several studies have shown that mindfulness therapy could be used to enhance life satisfaction, positive affect, gratitude, self-compassion (39, 42, 43). Moreover, lower sleep quality contributed to the lower psychological health QOL, confirming earlier reports showing a negative association between sleep quality and psychological health QOL (8).

In a recent umbrella review, Jahrami et al. (44) investigated the prevalence of psychological and behavioral symptoms among medical students. The authors found a substantial prevalence of symptoms, including sleep problems, stress, anxiety, depression, burnout, internet addiction, substance use, eating disorders, and suicidal thoughts. These problems, which seems arising from academic-related stressors, can have severe consequences such as poor academic performance, increased thoughts of dropping out of medical schools, and decreased professionalism (44). In a similar study, Marques et al. (45) explored the association between sleep quality and QOL in healthy young adults, showing that sleep quality is a significant predictor of QOL in all domains, with the exception of social relationships. Similarly, an earlier research showed that poor sleep quality was associated with reduced physical and psychological well-being (46). Although not investigated in our study, factors such as living alone (38) or living with guardians (47) and high caffeine intake (47) contribute to poor sleep quality and lower psychological health domain of QOL among undergraduate students. Taken together, sleep quality is a strong predictor of QOL, and sleep hygiene education might contribute to better sleep quality which may, to a certain degree, enhance the physical and psychological domains of QOL in college students.

We showed that PA directly and indirectly enhanced the psychological domain of QOL (through enhancing sleep quality). In this regard, the advantage of regularly practicing PA could be multifaceted. PA could contribute to maintaining a healthy body weight and increasing self-esteem (38), enhancing cardiac activity and emotion processing (4), reducing stress and anxiety (17), favoring good sleep quality and improving physical and mental health (17). Therefore, practicing PA will enhance the QOL of college students, which has also been reported by other studies (4, 17, 38, 48).

### Social relationships QOL

We found strong correlations between sleep quality, depression, stress, and the social relationships’ domain of QOL. Our findings align with those of Kent et al. (49) reporting a strong correlation between various social ties and sleep quality, with depression mediating the relationship between social support and the sleep quality of older college students (50). Indeed, social relationships are important for improving both physical and mental health, which also reduces stress levels (51). In this context, personal control closely links with social ties, plays a crucial role for positive health habits, mental, and physical health. Alsubaie et al. (52) investigated the impact of social support on the mental health and wellbeing of university students. They showed a strong correlation between higher social support from friends and family with (i) lower depression symptoms and (ii) higher psychological and social relationships QOL domains. Furthermore, they also observed that a deficiency of social support contributes to mental health issues. These findings indicate that lower levels of depression result in higher QOL within the psychological and social relationship domains. Our results differ from the literature as we observed a relatively low, but significant positive relationship between stress and social relationship’ QOL. Our study design does not allow assessing the reasons between the discrepancy of this result with the above-mentioned references, however, we speculate that this might be because the students constituting our study population might have ensured social engagement time at the expenses of their study’ time, resulting in a certain amount of stress. This deserves to be investigated by future studies.

### Environmental QOL

We noted that the impacts of depression and anxiety levels on environmental QOL exceeded those of sleep quality and physical activity. Indeed, environmental QOL is an important element affecting life expectancy and mental health (49, 50). This could be attributed to the fact that long-term exposure to a polluted environment can negatively impact individuals’ cognitive abilities and induce depression (53). Chang et al. (48) showed that access to green space induces higher physical QOL and increased levels of physical activity. Their study emphasized the significance of individual health behaviors, environmental factors, as well as perceived stress and sleep quality in influencing various domains of QOL. They also unveiled that satisfaction with public spaces exhibited a positive correlation with all domains of QOL. Furthermore, Chang et al. (48) have shown that the negative association of environmental quality with QOL, is mediated by perceived stress and poor sleep. These findings imply that interventions aiming at addressing these factors could potentially lead to enhancements in QOL.

## Strengths and limitations

Our study included a large international sample (*n* > 3,300) of college/university students from ~50 countries allowing to draw robust conclusions and the generalization of the outcome for the main results. Further, the study was conducted by an international network of university teachers working closely with students which favors a greater understanding of the academic-life challenge and the communication of the results to the targeted population. Within our study limitations, the use of a cross-sectional research design based on a survey cannot allow establishing causality and relies on subjective reporting that is subject to potential social desirability bias. Furthermore, our sample’s sex and continental allotment does not represent the world actual population distribution. The overrepresentation of women and both Africa and Europe continents, should be considered when interpreting our results. Additionally, the survey was conducted in three languages only (Arabic, English, French), and participation was limited to individuals who understand these languages, which may further influence the generalizability of our findings. Future research should consider using an experimental or longitudinal research design to examine the associations between sleep quality, psychological factors and QOL.

## Practical implication and recommendations

Several researchers suggest that efforts to improve the duration, efficiency, and overall sleep habits could have positive consequences on individuals’ well-being (54, 55). They suggest that sleep hygiene principles should not only be directed toward those suffering from sleep-related disorders but should also be extended to healthy young adults and students. Establishing a regular bedtime and wake-up routine can greatly enhance general well-being and productivity. Consistently following these sleep hygiene principles could improve sleep quality, increase energy levels, and enhance overall well-being (54). Our study showed that students might exhibit sleep problems, anxiety, and depression symptoms, highlighting the importance of promoting and supporting physical activity among university students to potentially improve various aspects of their QOL. Improving the QOL for university students requires a multifaceted approach that addresses sleep quality, mental health, and physical activity while promoting supportive environmental and social conditions. Such comprehensive strategies are vital for enhancing students’ overall well-being and academic performance, ultimately contributing to their success and satisfaction in their academic and personal lives.

## Conclusion

We elucidated the interrelations among sleep quality, mental health, and physical activity on different domains of QOL in college students. Our innovative results showed that environmental QOL was associated with both sleep quality and physical activity. The association between sleep quality and both physical and mental QOL implies a potential influence on subsequent health-related outcomes. Depression, stress and anxiety strongly affect the psychological health QOL. These findings emphasize the advantage of early identification of sleep and mental health problems in college students and to implement the appropriate preventive strategies. This, will inform the development of effective strategies to promote overall well-being in this population, hopefully improving their overall QOL and academic performance.

## Data availability statement

The raw data supporting the conclusions of this article will be made available by the authors, without undue reservation.

## Ethics statement

The studies involving humans were approved by the Ethics Committee of Qatar University (QU-IRB 1510-EA/21). The studies were conducted in accordance with the local legislation and institutional requirements. The participants provided their written informed consent to participate in this study.

## Author contributions

IM-C: Writing – review & editing, Writing – original draft, Visualization, Validation, Supervision, Software, Resources, Project administration, Methodology, Investigation, Funding acquisition, Formal analysis, Data curation, Conceptualization. AF: Writing – review & editing, Writing – original draft, Visualization, Validation, Supervision, Software, Resources, Project administration, Methodology, Investigation, Formal analysis, Data curation, Conceptualization. MR: Writing – review & editing, Writing – original draft, Visualization, Validation, Supervision, Software, Resources, Project administration, Methodology, Investigation, Formal analysis, Data curation, Conceptualization. JW: Writing – review & editing, Writing – original draft, Visualization, Validation, Resources, Methodology, Investigation, Formal analysis, Data curation, Conceptualization. UB: Writing – review & editing, Writing – original draft, Visualization, Validation, Resources, Methodology, Investigation, Formal analysis, Data curation, Conceptualization. MH: Writing – review & editing, Writing – original draft, Visualization, Validation, Investigation. RA-H: Writing – review & editing, Writing – original draft, Visualization, Validation, Investigation, Conceptualization. PS: Writing – review & editing, Writing – original draft, Visualization, Validation, Investigation, Data curation, Conceptualization. NR: Writing – review & editing, Writing – original draft, Visualization, Validation, Supervision, Methodology, Conceptualization. OH: Writing – review & editing, Writing – original draft, Visualization, Validation, Supervision, Methodology, Conceptualization.

## Footnotes

^1^ gdpr-info.eu
