# Supplementary material for: The relationship between quality of life, sleep quality, mental health, and physical activity in an international sample of college students: a structural equation modeling approach
Source: Front Public Health. 2024 Jul 10;12:1397924. doi: 10.3389/fpubh.2024.1397924 (PMC11266085; doi:10.3389/fpubh.2024.1397924)
Supplement: Supplementary file 1 [file Table_1.docx]

| **Tool/instrument** | **Arabic version** | **French version** | **English version** |
| --- | --- | --- | --- |
| **WHO (QOL)** | - Ohaeri JU, Awadalla AW. The reliability and validity of the short version of the WHO Quality of Life Instrument in an Arab general population. Ann Saudi Med. 2009;29(2):98–104. [10.4103/0256-4947.51790](https://doi.org/10.4103/0256-4947.51790) | - Baumann C, Erpelding ML, Régat S, Collin JF, Briançon S. The WHOQOL-BREF questionnaire: French adult population norms for the physical health, psychological health and social relationship dimensions. Rev Epidemiol Sante Publique. 2010 Feb 1;58(1):33–9. [10.1016/j.respe.2009.10.009](https://doi.org/10.1016/j.respe.2009.10.009) | - Skevington SM, Lotfy M, O’Connell KA. The World Health Organization’s WHOQOL-BREF quality of life assessment: Psychometric properties and results of the international field trial a Report from the WHOQOL Group. Qual Life Res. 2004 Mar;13(2):299–310. [10.1023/B:QURE.0000018486.91360.00](https://doi.org/10.1023/b:qure.0000018486.91360.00) |
| **PSQI** | - Suleiman KH, Yates BC, Berger AM, Pozehl B, Meza J. Translating the pittsburgh sleep quality index into Arabic. West J Nurs Res. 2010;32(2):250–68. [10.1177/0193945909348230](https://doi.org/10.1177/0193945909348230) | Blais FC, Gendron L, Mimeault V, Morin CM. Evaluation of insomnia: validity of 3 questionnaires. Encephale. 1997 Nov 1;23(6):447–53. | - Buysse DJ, Reynolds CF, Monk TH, Berman SR, Kupfer DJ. The Pittsburgh Sleep Quality Index: a new instrument for psychiatric practice and research. Psychiatry Res. 1989 May;28(2):193–213. [10.1016/0165-1781(89)90047-4](https://doi.org/10.1016/0165-1781(89)90047-4) |
| **ESS** | - Ahmed AE, Fatani A, Al-Harbi A, Al-Shimemeri A, Ali YZ, Baharoon S, et al. Validation of the Arabic version of the Epworth Sleepiness Scale. J Epidemiol Glob Health. 2014;4(4):297–302. [10.1016/j.jegh.2014.04.004](https://doi.org/10.1016/j.jegh.2014.04.004) | - Kaminska M, Jobin V, Mayer P, Amyot R, Perraton-Brillon M, Bellemare F. The Epworth Sleepiness Scale: Self-Administration Versus Administration by the Physician, and Validation of a French Version. Can Respir J. 2010;17(2):e27–34. [10.1155/2010/438676](https://doi.org/10.1155/2010/438676) | - Johns MW. A new method for measuring daytime sleepiness: the Epworth sleepiness scale. Sleep. 1991;14(6):540–5. [10.1093/sleep/14.6.540](https://doi.org/10.1093/sleep/14.6.540) |
| **ISI** | Suleiman, K. H., & Yates, B. C. (2011). Translating the insomnia severity index into Arabic. *Journal of Nursing Scholarship*, *43*(1), 49-53. [10.1111/j.1547-5069.2010.01374.x](https://doi.org/10.1111/j.1547-5069.2010.01374.x) | - Chahoud M, Chahine R, Salameh P, Sauleau EA. Reliability, factor analysis and internal consistency calculation of the Insomnia Severity Index (ISI) in French and in English among Lebanese adolescents. eNeurologicalSci. 2017 Jun 1;7:9–14. [10.1016/j.ensci.2017.03.003](https://doi.org/10.1016/j.ensci.2017.03.003) | - Bastien CH, Vallières A, Morin CM. Validation of the insomnia severity index as an outcome measure for insomnia research. Sleep Med. 2001;2(4):297–307. [10.1016/s1389-9457(00)00065-4](https://doi.org/10.1016/s1389-9457(00)00065-4) |
| **DASS 21** | - Ali AM, Ahmed A, Sharaf A, Kawakami N, Abdeldayem SM, Green J. The Arabic Version of The Depression Anxiety Stress Scale-21: Cumulative scaling and discriminant-validation testing. Asian J Psychiatr. 2017 Dec 1;30:56–8. [10.1016/j.ajp.2017.07.018](https://doi.org/10.1016/j.ajp.2017.07.018) | Sajeda Nahaboo (Thesis). Validation of the French Depression Anxiety Stress Scales (DASS-21) and predictors of depression in an adolescent Mauritian population. September 2015 <https://acesse.dev/Rthl5> | - Henry JD, Crawford JR. The short-form version of the Depression Anxiety Stress Scales (DASS-21): Construct validity and normative data in a large non-clinical sample. Br J Clin Psychol. 2005 Jun 1 ;44(2):227–39. [10.1348/014466505X29657](https://doi.org/10.1348/014466505x29657) |
| **IPAQ** | Helou, K., El Helou, N., Mahfouz, M., Mahfouz, Y., Salameh, P., & Harmouche-Karaki, M. (2017). Validity and reliability of an adapted Arabic version of the long international physical activity questionnaire. BMC public health, 18(1), 49. [10.1186/s12889-017-4599-7](https://doi.org/10.1186/s12889-017-4599-7) | - Meh K, Jurak G, Sorić M, Rocha P, Sember V. Validity and Reliability of IPAQ-SF and GPAQ for Assessing Sedentary Behaviour in Adults in the European Union: A Systematic Review and Meta-Analysis. Int J Environ Res Public Heal 2021, Vol 18, Page 4602. 2021 Apr 26;18(9):4602. [10.3390/ijerph18094602](https://doi.org/10.3390/ijerph18094602) | |

**Table S1: Different languages’ versions of the questionnaires used in the survey**
